# Effective Efficiency Advantage Assessment of Information Filter for Conventional Kalman Filter in GNSS Scenarios

**DOI:** 10.3390/s19183858

**Published:** 2019-09-06

**Authors:** Yanning Zheng, Siyou Wang, Shengli Wang

**Affiliations:** 1 College of Geomatics, Shandong University of Science and Technology, Qingdao 266590, China; 2 Ocean Science and Engineering College, Shandong University of Science and Technology, Qingdao 266590, China

**Keywords:** computational efficiency, Global Navigation Satellite System, information filter, Kalman filter

## Abstract

The Global Navigation Satellite System (GNSS) is a widely used positioning technique. Computational efficiency is crucial to applications such as real-time GNSS positioning and GNSS network data processing. Many researchers have made great efforts to address this problem by means such as parameter elimination or satellite selection. However, parameter estimation is rarely discussed when analyzing GNSS algorithm efficiency. In addition, most studies on Kalman filter (KF) efficiency commonly have defects, such as neglecting application-specified optimization and limiting specific hardware platforms in the conclusion. The former reduces the practicality of the solution, because applications that need such analyses on filters are often optimized, and the latter reduces its generality because of differences between platforms. In this paper, the computational cost enhancement of replacing the conventional KF with the information filter (IF) is tested considering GNSS application-oriented optimization conditions and hardware platform differences. First, optimization conditions are abstracted from GNSS data-processing scenarios. Then, a thorough analysis is carried out on the computational cost of the filters, considering hardware–platform differences. Finally, a case of GNSS dynamic differencing positioning is studied. The simulation shows that the IF is slightly faster for precise point positioning and much faster for the code-based single-difference GNSS (SDGNSS) with the constant velocity (CV) model than the conventional KF, but is not a good substitute for the conventional KF in the other algorithms mentioned. The real test shows that the IF is about 50% faster than the conventional KF handling code-based SDGNSS with the CV model. Also, the information filter is theoretically equivalent to and can produce results that are consistent with the Kalman filter. Our conclusions can be used as a reference for GNSS applications that need high process speed or real-time capability.

## 1. Introduction

The Kalman filter is a widely used data-processing tool in many areas of engineering, including positioning and navigation [1]. Due to the diversity and complexity of real engineering problems, the conventional Kalman filter (KF) is not always able to get acceptable results, which necessitates improved versions for different purposes. There are some examples: for nonlinear models, the extended Kalman filter, particle filter, and cubature filter have been proposed and applied in Global Navigation Satellite System (GNSS) precise point positioning (PPP) [2,3]; for the inaccuracy of the stochastic model and coarse error, the adaptive Kalman filter, H_∞_ filter, and maximum correntropy Kalman filter can be applied in algorithms such as an integrated GNSS/inertial navigation system (INS) [4]; the decentralized Kalman filter and Kalman consensus filter are used to handle distributed computing problems, such as underwater cooperative navigation [5]; and several square root filters have been proposed for better numerical performance, and work well in many scenarios [6,7].

For some engineering realms, computational complexity gets as much attention as other issues, such as nonlinear properties and numerical performance. The generation of real-time state space representation (SSR) products in International GNSS Service (IGS) analysis centers is an example. The existing literature on computational optimization and assessment can be mainly classified into three problems: GNSS network data processing, fast satellite selection algorithm, and fast ambiguity resolution. For fast and real-time GNSS network data processing, Ge et al. proposed a strategy based on parameter elimination, and processing time reduced to less than one-third compared to previous methods [8]. In a study by Gong et al., the processing time of multi-GNSS network data was reduced by almost two orders by employing blocked QR factorization algorithms [9]. QR factorization is a decomposition process that for matrix A, we have A=QR, where Q and R are an orthogonal matrix and upper triangular matrix, respectively [10]. The key idea of the study is that reorganizing algorithms in the form of large granularity operations can reduce data copying between registers, cache, and memory, and reduce time cost. Barbu et al. used QR factorization and an improved matrix reduction procedure to lessen the computational time for GINS software [7]. The authors discussed the proposed method in detail, but did not do much analysis on computational cost improvement. Fu et al. studied real-time clock estimation and its quality control, with some discussion of processing time cost to ensure real-time performance [11]. However, since the major concern of the article is clock estimation, the authors did not compare the computational cost performance with previous approaches. For the satellite selection method, since it is sometimes applied to reduce computational burden, its own computational cost will be crucial. Articles by Liu et al. and Meng et al. proposed fast satellite selection approaches, but both sacrificed optimality slightly [12,13]. For fast ambiguity resolution, Jazaeri et al. used lattice theory and demonstrated obvious optimization of the time cost [14]. Baselga discussed an ambiguity-free method, with advantages including shorter running time [15]. These studies brought many useful methods to improve computational cost from multiple aspects, but the computational cost of filters used in positioning and navigation algorithms is not mentioned much.

There are some studies on the computational cost performance of different Kalman filters. Bierman compared five filters based on their computational cost, and concluded that the information filter (IF) is faster than the KF, and so is its square root version [16]. However, the test covered only a few situations, and was based on one old-model computer. Modern computers have many features that old-model computers don’t have, which makes the conclusion of this study not so suitable for modern computers. Mendel made a thorough analysis of the computational complexity and storage requirements of the Kalman filter [17]. The author analyzed individual arithmetic operations, but only demonstrated two specific examples for the overall time cost. An article by Bierman and a book by Grewal et al. mentioned that the decorrelation of observations can unify algorithms with correlated observations and with independent observations in the analysis of computational efficiency [16,18]. Since such decorrelation can be achieved by LDLT decomposition, this statement will hold only if the computational advantage of a diagonal observation covariance matrix over a nondiagonal one is much greater than the cost of the LDLT decomposition. Thus, algorithms with correlated observations and those with independent observations cannot be unified in computational cost analysis. Then, optimizations based on observation independencies are algorithm-specified. From the literature above, one can determine the following defects: (1) the arithmetic operation time used in these studies may not suit modern 64-bit computers; (2) the differences between hardware platforms were not seriously taken into account; and (3) algorithm-specific optimization should be considered for practicality, because computational cost is always tightly related to optimization.

In this paper, the computational improvement of replacing the KF with the IF in positioning and navigation scenarios is studied. Hardware platform differences are taken into account to alleviate any platform limitation of the results and conclusions, and algorithm-specified optimizations are considered for better practicality. The related GNSS positioning and navigation scenarios and their descriptions are as follows:

1. Precise point positioning (PPP): PPP uses pseudo-range and carrier-phase observations from a single GNSS receiver, precise satellite orbit, and clock products, and refined systematic error models to achieve high-precision positioning [19]. PPP usually uses the KF as the parameter estimator. The ionosphere-free PPP (IFPPP) algorithm uses an ionosphere-free combination of observations, with estimated parameters of position corrections in the x, y, and z directions, a wet component of zenith tropospheric delay, a receiver clock bias, and ambiguities with ionospheric combination [19]. The uncombined PPP (UPPP) algorithm uses raw observations, with estimated parameters of conventional PPP plus slant ionospheric delay parameters [20].
2. GNSS difference positioning (DGNSS): DGNSS uses pseudo-range or carrier-phase observations from two receivers to determine their coordinate difference [19]. Single-differenced DGNSS (SDGNSS) only applies the difference between two receivers; double-differenced DGNSS (DDGNSS) applies the difference both between two receivers and between a reference satellite and other satellites. Pseudo-range (code-based) DGNSS (code-DGNSS) traditionally only uses pseudo-range for position estimation, and carrier-phase DGNSS (real-time kinematic, RTK) uses carrier-phase observations. In this paper, DGNSS is also combined with a constant velocity (CV) model and Doppler observations. When a receiver is mounted to a vehicle such as an automobile, it is possible to apply specific dynamic model restriction in the parameter estimation process. Constant acceleration (CA) and constant velocity (CV) models are two examples [21]. The CA model assumes the target performs uniformly accelerated motion between two epochs, and the CV model assumes the target performs uniform motion between two epochs. Such a model exploits the advantage of multisource information for better precision and robustness, and requires KF as the data-processing tool. With proper process noise, these dynamic models will at least not degrade the positioning results, and the closer the real dynamic characteristics to the given model, the better the solution. Since the CV model introduces velocity parameters into the filter, information about velocity is required to make the model really effective, and Doppler observations exactly suit the need. So, the test will use Doppler observations along with pseudo-range observations. This special code-DGNSS model is called code-DGNSS with CV in this paper.
3. GNSS/INS (inertial navigation system) integrated navigation: This type of algorithm integrates results from GNSS and INS. Here, the GNSS algorithm in use can be either undifferenced or differenced, leading to algorithms including PPP/INS, RTK/INS, and so on. Further, there are two major integration methods: loosely coupled (LC) integrates position solutions from GNSS and INS directly, and tightly coupled (TC) integrates GNSS observations and observations derived from INS position solutions [22]. The Kalman filter is used to conduct such integration.

The rest of this paper is organized as follows: basic definitions of the filters; algorithm characteristics and corresponding filter optimizations; computational cost polynomials formulation; theoretical analyses; case study; and conclusions.

## 2. Basic Definitions of the Filters

In this section, the equations of KF and IF are provided, and the related variables and symbols are defined.

### 2.1. Conventional Kalman Filter

Assume the state space model of the stochastic system is:

(1) $$\{\begin{array}{c} x_{k+1}=Fx_{k}+w\\ L=Bx_{k+1}+v \end{array}$$

and w and v are independent Gaussian sequences with zero means:

(2) $$\{\begin{array}{c} E\left(w_{j}\right)=0,\;E\left(w_{j}w_{k}^{T}\right)=W_{j}\delta_{j,k}\\ E\left(v_{j}\right)=0,\;E\left(v_{j}v_{k}^{T}\right)=R_{j}\delta_{j,k}\\ E\left(w_{j}v_{k}^{T}\right)=0 \end{array}$$

where x is the system state vector, F is the state transition matrix, w is the process noise vector, L is the observation vector, B is the observation matrix, v is the observation noise vector, W is the process noise covariance matrix, R is the observation noise covariance matrix, *k* and *k*+1 indicate two adjacent epochs, *j* and *k* are two arbitrary epochs, and *E* is the mathematical expectation operator. The estimated state value and its covariance can be calculated by KF with the following equations [18,23]:

(3) $${\bar{x}}_{k+1}=F{\hat{x}}_{k}$$

(4) $${\bar{Q}}_{k+1}=F{\hat{Q}}_{k}F^{T}+W$$

(5) $$K={\bar{Q}}_{k+1}B^{T}{\left(B{\bar{Q}}_{k+1}B^{T}+R\right)}^{-1}$$

(6) $${\hat{x}}_{k+1}={\bar{x}}_{k+1}+K\left(L-B{\bar{x}}_{k+1}\right)$$

(7) $${\hat{Q}}_{k+1}=\left(E-KB\right){\bar{Q}}_{k+1}$$

where K is the Kalman filter gain matrix. The bars over some variables mean that they are predicted based on historical information, and the carets over some variables mean that they are estimated values based on historical information and observations of the current epoch. Equations (3) and (4) constitute the time update step of the filters. Equations (5)–(7) constitute the measurement update step of the filters.

### 2.2. Information Filter

The IF is a kind of KF. It uses information vector S=Ix and information matrix $I=Q^{-1}$ to replace parameter vector x and parameter covariance matrix Q used in KF [24]. It was first proposed for spacecraft navigation to handle the lack of initial state information in backward filtering [25]. For the same state space model of Equation (1), IF can be updated with the following equations [24,25]:

(8) $$M={\left(F^{T}+{\hat{I}}_{k}F^{-1}W\right)}^{-1}$$

(9) $${\bar{I}}_{k+1}=M{\hat{I}}_{k}F^{-1}$$

(10) $${\bar{S}}_{k+1}=M{\hat{S}}_{k}$$

(11) $${\hat{I}}_{k+1}={\bar{I}}_{k+1}+B^{T}R^{-1}B$$

(12) $${\hat{S}}_{k+1}={\bar{S}}_{k+1}+B^{T}R^{-1}L$$

Equations (8)–(10) constitute the time update step. Equations (11) and (12) constitute the measurement update step. I and S can be transformed to Q and x, or conversely as Equations (13) and (14), if I is invertible:

(13) $$\{\begin{array}{c} Q=I^{-1}\\ x=QS \end{array}$$

(14) $$\{\begin{array}{c} I=Q^{-1}\\ S=Ix \end{array}$$

Since the computer implementations of IF often store and update I and S, Equation (13) is used only if epoch solutions are needed, and Equation (14) is used only for the filter initialization.

It should be emphasized that IF is derived from KF directly and is theoretically equivalent to KF. Mutambara made a detailed comparison in his book, and concluded that the difference between their solutions is merely numerical error [24]. Thus, all of our discussions are based on their solution equivalence.

## 3. Algorithm Characteristics and Corresponding Filter Optimizations

Efficiency assessment is usually related to optimization. Thus, taking optimizations specified by algorithm characteristics into account will make the analysis of efficiency more practical for application. In this section, several GNSS data-processing algorithms are discussed to find out their characteristics and possible filter optimizations.

### 3.1. Characteristics of GNSS Data-Processing Algorithms

Data-processing algorithms have different characteristics, which have an influence on the computational cost. Here, these characteristics are abstracted as factors for further discussion, by inspecting real GNSS positioning and navigation algorithms. Essentially, these factors decide what operations can be removed or replaced with simpler ones in each filter update cycle. Please note that these algorithms are discussed from three aspects: (1) solution requirement, (2) dynamic model, and (3) observation model.

1. Undifferenced GNSS algorithms: (1) For GNSS applications for static positioning purposes, only the parameter estimation and covariance of the last epoch is necessary, while all epoch solutions are needed for kinematic positioning. (2) In many undifferenced GNSS algorithms, matrices F and W are invariant to epochs. (3) Undifferenced GNSS algorithms use undifferenced observations; thus, the R matrices are diagonal.
2. DGNSS: (1) Since these algorithms are widely used in kinematic positioning, the position solution from each epoch is often of interest. (2) For DGNSS using a constant velocity (CV) or constant acceleration (CA) dynamic model, matrices F and W are invariant in epochs, unless the stability of the clock bias cannot be guaranteed (such as with clock jump). (3) The R matrices can be either diagonal (single-differenced) or nondiagonal (double-differenced) in these algorithms.
3. GNSS/INS integrated navigation: (1) Similar to RTK algorithms, these algorithms also need each epoch solution. (2) Due to the intricate nonlinear dynamic model of the INS, matrix F is generally determined by the current position, velocity, and attitude, and is variant to epoch. (3) Impacted by the sophisticated inertial measurement unit (IMU) dynamic model, matrix R is nondiagonal.

From the discussions above, it is easy to notice the variety of algorithm characteristics. For these characteristics, three filter factors are summarized in Table 1, which will be essential to the subsequent discussions. Please note that two additional options are considered, 2B and 3A. These options do not correspond to any algorithms in this paper, but appear in other scenarios, and can make the analysis more comprehensive. For example, option 2B could correspond to a simple population growth model in the form of $x_{k+1}=\left(1+f\right)x_{k}$, where *f* is the growth rate; option 3A could correspond to a simplified GNSS network adjustment algorithm with fixed B and R matrices, since it can be tempting to lose a little optimality and make it possible to provide a real-time network solution.

### 3.2. Filter Optimizability

With the table above, we can analyze the filter Equations (3)–(14) to determine the optimizable arithmetic operations using different options of each factor. Since the last options of each factor correspond to unoptimized situations, they will be used as references.

Factor 1: For KF, obtaining the epoch solutions Q and x is necessary for the filter update. Thus, even if the epoch solutions are not needed, they will still be obtained, and no calculation can be simplified. For IF, Equation (13) can be omitted if epoch solutions are not required, which can reduce some computational cost.

Factor 2: For KF, matrices F and W are used only in Equation (4). Assume that option A is selected and color is applied to Equation (4) as in Equation (15), where red indicates quantities that vary with epochs, and green indicates quantities that are constant. Since every computational step of this equation involves variable quantities directly or indirectly, no calculation can be simplified. Assume that option B is selected. As Equation (4) does not involve an inversion of F, the computational cost will remain unchanged. For IF, matrices F and W are used in Equations (8) and (9). Assume that option A is selected and color is applied to Equations (8) and (9) as Equations (16) and (17). Here, $F^{-1}W$ and $F^{-1}$ can be calculated before the filter starts, and the computational cost of a single filter update procedure can be reduced. Assume that option B is selected. The inversion of F will become easy and fast when F is diagonal, which means less computational cost:

(15) $${\bar{Q}}_{k+1}=F{\hat{Q}}_{k}F^{T}+W$$

(16) $$M={\left(F^{T}+{\hat{I}}_{k}F^{-1}W\right)}^{-1}$$

(17) $${\bar{I}}_{k+1}=M{\hat{I}}_{k}F^{-1}$$

Factor 3: For KF, matrices B and R are used in Equations (5)–(7). Assume that option A is selected and color is applied to Equations (5)–(7) as in Equations (18)–(20). Since every computational step of these equations involves variable quantities directly or indirectly, no calculation can be simplified. Assume that option B is selected. As these three equations do not involve an inversion of R, the computational cost will remain unchanged. For IF, matrices B and R are used in Equations (11) and (12). Assume that option A is selected and color is applied to Equations (11) and (12) as in Equations (21) and (22). Here, the calculation of $B^{T}R^{-1}B$ in Equation (21) and $B^{T}R^{-1}$ in Equation (22) can be done beforehand and removed from the filter update of each epoch. Assume that option B is selected. The inversion of R will become easy and fast when R is diagonal, which means less computational cost:

(18) $$K={\bar{Q}}_{k+1}B^{T}{\left(B{\bar{Q}}_{k+1}B^{T}+R\right)}^{-1}$$

(19) $${\hat{x}}_{k+1}={\bar{x}}_{k+1}+K\left(L-B{\bar{x}}_{k+1}\right)$$

(20) $${\hat{Q}}_{k+1}=\left(E-KB\right){\bar{Q}}_{k+1}$$

(21) $${\hat{I}}_{k+1}={\bar{I}}_{k+1}+B^{T}R^{-1}B$$

(22) $${\hat{S}}_{k+1}={\bar{S}}_{k+1}+B^{T}R^{-1}L$$

From the discussion above, we can see that, due to its computational properties, the KF happens to gain no computational cost benefit from different factor options. For each factor of a given algorithm, when the factor option A (for factors 1, 2, and 3) or B (for factors 2 and 3) is met, some computation in the filter update procedures of IF can be omitted or simplified. These optimizations of IF are summarized in Table 2.

Please note that the options of different factors are independent. For example, the options of factor 2 involve matrices F and W, and the options of the other two factors do not involve these two matrices. This makes the analysis easier, because the computational cost of every combination of these factor options can be expressed by the same group of basic computational cost functions. Also, for the convenience of description, the factor option combines with options $a_{1}$, $a_{2}$, and $a_{3}$ for factors 1, 2, and 3, respectively, where $a_{i}\in \left\{A,B,C\right\}$ will be called factor combination $a_{1}a_{2}a_{3}$.

## 4. Computational Cost of Polynomial Formulation

The computational cost of the filters is analyzed in a very straightforward way: The filter update procedures consist of matrix operations, which are addition (including subtraction), multiplication, and the inversion of specific magnitudes. The matrix operations consist of scalar operations, which are addition (including subtraction), multiplication, and division. In this section, the computational complexities of the matrix operations are first expressed in the form of scalar operation number polynomials; then, equations to assemble polynomials for given factor combinations from a group of basic polynomials are put forward; finally, this group of basic polynomials is given.

Table 3 shows the polynomials that represent the computational cost of matrix operations. Please note that two matrix inversion algorithms are included. The polynomials of addition and multiplication can be obtained easily from the definition of matrix operations. The polynomials of LU and LDLT decompositions can be obtained from their computational implementations, which can be found in Sauer’s book and Sun’s article [10,26]. LU decomposition calculates the lower triangular matrix L and upper triangular matrix U for a given matrix A in which A=LU; and LDLT decomposition calculates the lower triangular matrix L and diagonal matrix D for a given matrix A in which $A=LDL^{T}$.

$P_{typ,ope,a_{1},a_{2},a_{3}}\left(n,t\right)$ is used to refer to the polynomial of the filter type *typ* and scalar operation *ope*, with option combination $a_{1}a_{2}a_{3}$ of Table 2, where typ∈{KF,IF}; ope∈{A,M,D} (*A* for addition, *M* for multiplication, and *D* for division); $a_{1}\in \left\{A,B\right\}$, $a_{2}\in \left\{A,B,C\right\}$, $a_{3}\in \left\{A,B,C\right\}$; *n* is the observation number; and *t* is the dimension of the system state. The values and compositions of *n* and *t* for general GNSS algorithms are discussed in Section 5.4.

Since different factor options have no effect on the computational cost of KF, we have:

(23) $$P_{KF,ope,a_{1,i},a_{2,i},a_{3,i}}\left(n,t\right)=P_{KF,ope,a_{1,j},a_{2,j},a_{3,j}}\left(n,t\right)$$

for two arbitrary factor combinations, $a_{1,i}a_{2,i}a_{3,i}$ and $a_{1,j}a_{2,j}a_{3,j}$. So, one polynomial is enough for the KF.

For the IF, due to the independencies between options of different factors, Equation (24) is used to obtain the polynomial of arbitrary factor combinations $a_{1}a_{2}a_{3}$, where ${P^{\prime }}_{IF,ope,i,a_{i}}\left(n,t\right)$ is the polynomial representing the additional scalar operation number of option $a_{i}$ relative to option A of filter factor *i*.

(24) $$P_{IF,ope,a_{1},a_{2},a_{3}}\left(n,t\right)=P_{IF,ope,A,A,A}\left(n,t\right)+\sum_{i=1}^{3}{P^{\prime }}_{IF,ope,i,a_{i}}\left(n,t\right)$$

With Table 2 and Table 3, we can assemble every part on the right-hand side of Equation (24) by recording matrix operations and accumulating their polynomials. Please note that $F^{-1}$, where **F** is asymmetrical, uses LU decomposition, and other matrix inversions use LDLT decomposition. The inversion on the diagonal matrix is done by obtaining reciprocals of diagonal entries. These polynomials are summarized in Table 4, Table 5 and Table 6.

## 5. Theoretical Analyses

In this section, the test of scalar operation time cost conducted on multiple hardware platforms is discussed first to gain insight into the differences between platforms; then, the computational cost difference between KF and IF is analyzed, considering different factor combinations, observation numbers, and parameter numbers. Then, another test is carried out to validate our analysis on various platforms; and finally, another comparison of the two filters is carried out from the perspective of GNSS algorithms.

### 5.1. Test of Scalar Operation Time Cost

To measure the time cost of an individual scalar operation, a C++ program is designed. This program can do a single arithmetic operation $2\times 10^{9}$ times and calculate the duration. This pseudo-code has several features to ensure its effectiveness: (1) it operates only three variables when measuring time cost, which can reduce memory access; (2) it performs the same set of operations (addition, multiplication, division, and empty loop) 10 times to smooth the results; and (3) by introducing a test on an empty loop, the code takes irrelevant operations into account, such as loop jump and system time query.

Due to the complexity and variety of hardware platforms, the time cost test results from different platforms are not always consistent with each other. This greatly limits the study of computational efficiency. To determine the diversity, a group of various X64 hardware platforms were selected to run the test. Their basic information and test results are shown in Table 7.

**Figure 1:**
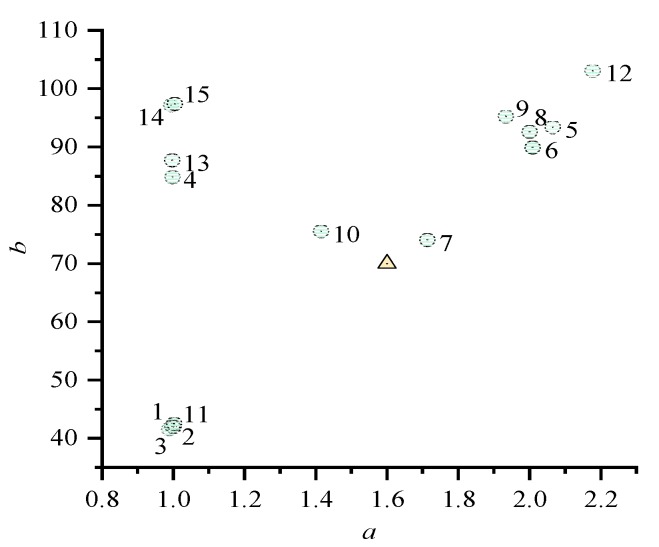
Ratio between time costs of multiplication and addition. Bottom axis: *a*; left axis: *b*.

The time costs of multiplication and division are divided by those of addition to obtain the time–cost ratios in the form of 1:a:b, where:

(25) $$a=\frac{\mathrm{dtM}-\mathrm{dtEmpty}}{\mathrm{dtA}-\mathrm{dtEmpty}}$$

(26) $$b=\frac{\mathrm{dtD}-\mathrm{dtEmpty}}{\mathrm{dtA}-\mathrm{dtEmpty}}$$

and the ratios are plotted in Figure 1. In Figure 1, the x and y axes are *a* and *b*. The circles with numbers correspond to the test results in Table 7. The ranges of *a* and *b* are approximately [1,2.2] and [40,100], respectively. Thus, a reference ratio point 1:1.6:70 is selected by minimizing the maximum differences with the circles on *a* or *b*. The triangle represents this reference ratio point, and the assessment is discussed in the next subsection. It shows that multiplication consumes about one to two times more time than addition, and division consumes about 40–100 times more time than addition.

### 5.2. Time Cost Analysis

**Figure 2:**
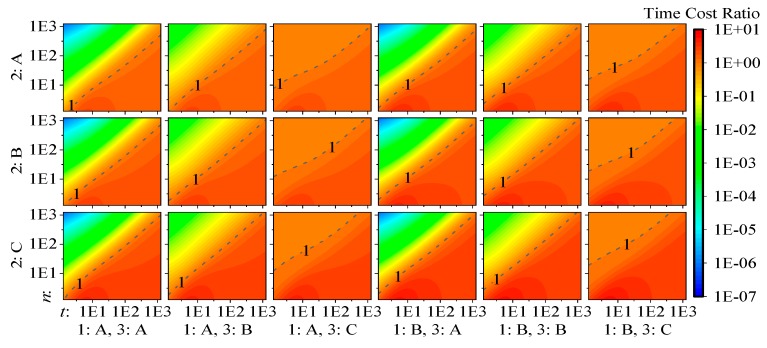
Computational cost ratio $t_{IF}/t_{KF}$.

Using the selected ratio set $1:a^{0}:b^{0}=1:1.6:70$ to assemble linear combinations of the polynomials from Table 4 through Table 6, computational cost polynomials for each factor combination and both filters are generated, and the ratio $t_{IF}/t_{KF}$ for parameter number and observation number ranging from 1 to 1000 is calculated. The results are plotted in Figure 2. Please note that both the x and y axes and the z axis (color) are in log10 scale. Arranging color in a linear scale compresses the ratios when the IF is faster than the KF into the range of [0,1], but a log10 scale can solve this problem.

From the plot, we can make the following conclusions:

1. From all 18 panels, we can see that the properties of the observational model affect the relative computational efficiency performance most obviously when n>t is satisfied, but the impacts of different types of dynamic models or whether epoch solutions are required are not very distinguishable. This can be explained by the following. (a) When n>t holds, R is larger than M and F, and its inversion dominates the overall computational cost. (b) When n<t holds, although the complexities of $F^{-1}$ and $I^{-1}$ depend on the corresponding filter characteristics and seem to influence the computational cost, the inversion in the computation of M, which uses inefficient LU decomposition and has invariant complexity to filter characteristics, dominates the overall computational cost and suppresses the impact of the dynamic model and the requirement of epoch solutions.
2. By comparing the panels of 3:A and 3:B with the panels of 3:C, we see that when the observational model is fixed or when R is diagonal, the IF shows much less time cost than the KF when n>t. Since the time cost differences are so large in such a situation, replacing the KF with the IF can reduce the time duration of the filter update to be almost negligible, but when n<t is satisfied, there is no big difference when using both filters.
3. From the panels of 3:C, we see that, for algorithms with a changeable, nondiagonal matrix R, the IF has no computational efficiency advantage, but it will not be obviously slower than the KF, either.
4. The decorrelation process in some studies, which is needed only when option C of factor 3 is satisfied, generally can be achieved by an n×n LDLT decomposition on matrix R, and enables option C of factor 3 to be handled as option B [16,18]. However, since the major computational advantage of option B compared with C is the omission of the n×n LDLT decomposition on R, the conclusions in those studies may not hold when R is nondiagonal. This can be proved by comparing the panels of 3:B with the panels of 3:C.

### 5.3. Applicability Assessment

**Figure 3:**
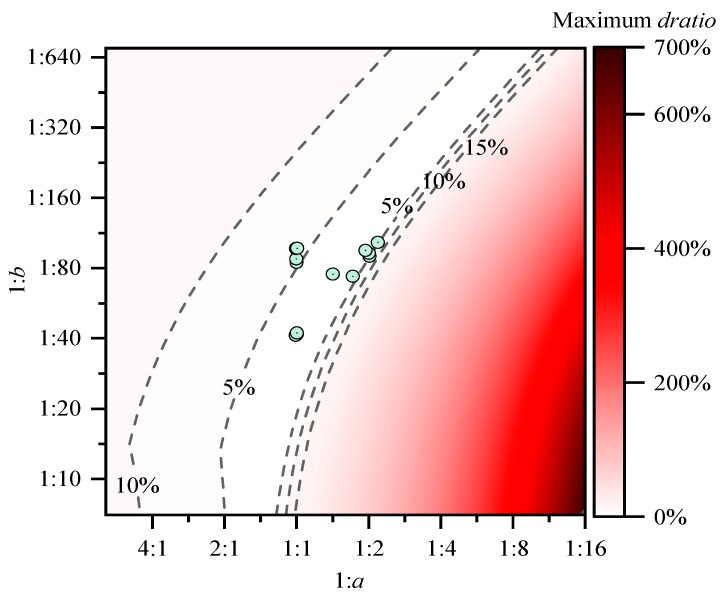
Maximum relative error of replacing the arbitrary ratio set 1:a:b with $1:a^{0}:b^{0}=1:1.6:70$.

Our analysis is still based on a given platform assumption that the time–cost ratio between addition, multiplication, and division is 1:1.6:70; thus, it is important to determine the maximum error *dratio* of replacing a real value from hardware platforms with the given result. In this subsection, the assessment is carried out by (1) giving a scalar operation time–cost ratio set at $1:a^{0}:b^{0}=1:1.6:70$; (2) giving another ratio set 1:a:b; (3) for 1≤t≤10000, 1≤n≤10000, and all 18 factor combinations, searching for the largest relative error (*dratio*) as Equations (27)–(29); and (4) plotting the largest error as a function of *a* and *b*. Figure 3 shows the result.

(27) $$dratio=\frac{\log_{10}\left(ratio\left(a_{0},b_{0}\right)\right)-\log_{10}\left(ratio\left(a,b\right)\right)}{\log_{10}\left(ratio\left(a,b\right)\right)}$$

(28) $$ratio\left(x,y\right)=\frac{t_{IF}\left(1,x,y\right)}{t_{KF}\left(1,x,y\right)}$$

(29) $$t_{typ}\left(x,y,z\right)=xP_{typ,A,a_{1},a_{2},a_{3}}\left(n,t\right)+yP_{typ,M,a_{1},a_{2},a_{3}}\left(n,t\right)+zP_{typ,D,a_{1},a_{2},a_{3}}\left(n,t\right)$$

Figure 3 shows that the ratio sets of all the platforms we tested previously can be replaced with $1:a^{0}:b^{0}=1:1.6:70$, with maximum relative errors less than around 10%. Since efficiency analyses are often biased by multiple factors that are hard to control and quantify, such as thermal dissipation, and the results differ from time to time, such a relative error upper bound is acceptable and will not affect the overall conclusions.

### 5.4. Time–Cost Ratio Prediction on General GNSS Positioning and Navigation Algorithms

**Figure 4:**
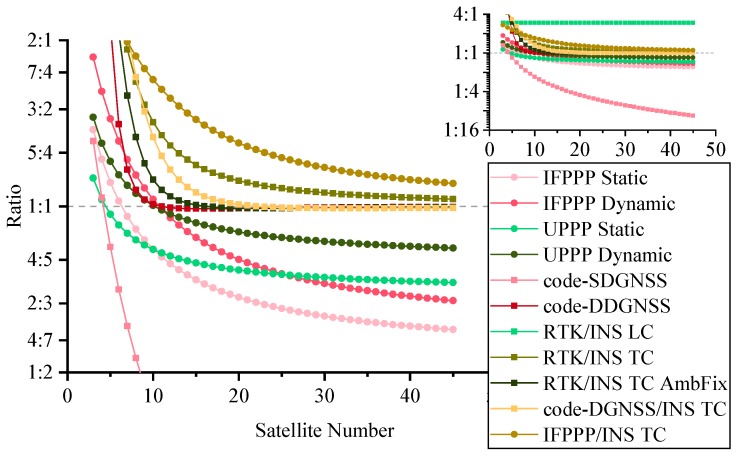
Computational cost ratio $t_{Info}/t_{Kal}$ for GNSS algorithms.

To optimize the computational complexity of general GNSS positioning and navigation algorithms, we can analyze the result from the last subsection to determine which is the fastest filter for a given algorithm. Table 8 shows the compositions and numbers of parameters and observations in GNSS algorithms, wherLU e s stands for satellite number. Table 9 shows the number of parameters, number of observations, and factor combination of each considered GNSS algorithm, where the dynamic feature of the position parameter is achieved by setting the proper process noise. GNSS systems in use are the Global Positioning System (GPS), BeiDou Navigation Satellite System (BDS), Galileo, and Global’naya Navigatsionnaya Sputnikovaya Sistema (GLONASS), and dual-frequency observations are used in all algorithms. Figure 4 demonstrates the variations of computational cost ratio to number of satellites, ranging from 3 to 45.

From the plot, we can see that:

1. The IF is slower than the KF or at the same level for algorithms with differencing between satellite and IMU data. This is mostly because these algorithms use the nondiagonal variant matrix R. For other algorithms, such as undifferenced PPP and code-SDGNSS, the IF is a good choice for replacing the KF and optimizing the running time.
2. The RTK/INS loosely coupled algorithm has a fixed ratio, because raw GNSS observations are not directly used.
3. Code-SDGNSS has an outstanding curve among all algorithms. This is because it does not use carrier-phase observations and correlated observations.
4. The computational difference between whether or not epoch solutions are required is hardly distinguishable in Figure 2; it is more obvious in Figure 4. Taking 20 satellites as an example, if the filter outputs a solution at every epoch, IF can save 22% and 11% computational cost compared with KF for IFPPP and UPPP, respectively. If the goal of data processing is the final position solution of the fixed station, this computational reduction will be 33% and 24% for IFPPP and UPPP, respectively.

## 6. Case Study

To verify the consistency between the analyses so far and filter performance in real data-processing scenarios, a case study based on the GNSS pseudo-range dynamic positioning algorithm with the constant velocity (CV) model is conducted. Generally, undifferenced and double-differenced (DD) algorithms are used in applications more than single-differenced (SD) algorithms. Compared with the SD model, the DD model has fewer observations and parameters, and thus is more computationally efficient using conventional KF; also, the DD model is important for integer ambiguity resolution. Our test will emphasize the correlation between DD observations, and show that SD pseudo-range positioning, which uses uncorrelated observations, is much more efficient when using the IF. Aside from the SD model, this section also involves the CV model and Doppler observations. With proper process noise, dynamic models such as CV and CA will at least not degrade the results of dynamic positioning, and the closer the real dynamic characteristics to the given model, the better the solution. Since the CV model introduces velocity parameters into the filter, information about velocity is required to make the model really effective, and Doppler observations exactly suit the need. So, the test uses Doppler observations along with pseudo-range observations. In this section, the GNSS single-differenced/double-differenced dynamic positioning algorithm with the CV model is briefly introduced; then, the details of the test are provided, and finally, the test result is studied.

### 6.1. GNSS Differencing Positioning Algorithm Using Pseudo-Range and Doppler Observations

The linearized observation equations of GNSS pseudo-range and Doppler observations are:

(30) $$\left\{ \begin{array}{l} \rho_{r1,s1}+v_{\rho ,r1,s1}=\frac{x_{r1}^{0}-x_{s1}}{R_{r1,s1}^{0}}dx_{r1}+\frac{y_{r1}^{0}-y_{s1}}{R_{r1,s1}^{0}}dy_{r1}+\frac{z_{r1}^{0}-z_{s1}}{R_{r1,s1}^{0}}dz_{r1}+c\cdot dt_{r1}-c\cdot dt_{s1}+R_{r1,s1}^{0}+\Delta other\\ D_{r1,s1}+v_{D,r1,s1}=\frac{{\dot{x}}_{r1}^{0}-{\dot{x}}_{s1}}{V_{r1,s1}^{0}}d{\dot{x}}_{r1}+\frac{{\dot{y}}_{r1}^{0}-{\dot{y}}_{s1}}{V_{r1,s1}^{0}}d{\dot{y}}_{r1}+\frac{{\dot{z}}_{r1}^{0}-{\dot{z}}_{s1}}{V_{r1,s1}^{0}}d{\dot{z}}_{r1}+c\cdot d{\dot{t}}_{r1}-c\cdot d{\dot{t}}_{s1}+V_{r1,s1}^{0}+\Delta other \end{array}\right.$$

where *ρ* is pseudo-range observations; *D* is Doppler observations; *v* is observation error; $\left[x_{r1},y_{r1},z_{r1}\right]$ and $\left[x_{s1},y_{s1},z_{s1}\right]$ are the positions of receiver *r*1 and satellite *s*1, respectively; $\left[{\dot{x}}_{r1},{\dot{y}}_{r1},{\dot{z}}_{r1}\right]$ and $\left[{\dot{x}}_{s1},{\dot{y}}_{s1},{\dot{z}}_{s1}\right]$ are the velocity of receiver *r*1 and satellite *s*1, respectively; $R_{r1,s1}=\sqrt{{\left(x_{r1}-x_{s1}\right)}^{2}+{\left(y_{r1}-y_{s1}\right)}^{2}+{\left(z_{r1}-z_{s1}\right)}^{2}}$ is the distance between the satellite and the receiver; $V_{r1,s1}=\sqrt{{\left({\dot{x}}_{r1}-{\dot{x}}_{s1}\right)}^{2}+{\left({\dot{y}}_{r1}-{\dot{y}}_{s1}\right)}^{2}+{\left({\dot{z}}_{r1}-{\dot{z}}_{s1}\right)}^{2}}$ is the relative velocity between the satellite and the receiver; *c* is light speed in a vacuum; $dt_{r1}$ and $dt_{s1}$ are the clock offsets of receiver *r*1 and satellite *s*1, respectively; $\Delta {\dot{t}}_{r1}$ and $\Delta {\dot{t}}_{s1}$ are the clock drifts of receiver *r*1 and satellite *s*1, respectively; Δother denotes all other systematic errors not appearing in the equations; and ${\left(*\right)}^{0}$ indicates the approximate value of (∗) and also where it is linearized.

For two receivers, *r*1 and *r*2, if *r*2 is fixed on the ground, we have the single-differenced (SD) equations:

(31) $$\{\begin{array}{c} \Delta \rho_{s1}+\Delta v_{\rho ,s1}=\frac{x_{r1}^{0}-x_{s1}}{R_{r1,s1}^{0}}\Delta dx+\frac{y_{r1}^{0}-y_{s1}}{R_{r1,s1}^{0}}\Delta dy+\frac{z_{r1}^{0}-z_{s1}}{R_{r1,s1}^{0}}\Delta dz+c\cdot \Delta dt_{r1,r2}+\Delta R_{s1}^{0}+\Delta other\\ \Delta D_{s1}+\Delta v_{D,s1}=\frac{{\dot{x}}_{r1}^{0}-{\dot{x}}_{s1}}{V_{r1,s1}^{0}}d{\dot{x}}_{r1}+\frac{{\dot{y}}_{r1}^{0}-{\dot{y}}_{s1}}{V_{r1,s1}^{0}}d{\dot{y}}_{r1}+\frac{{\dot{z}}_{r1}^{0}-{\dot{z}}_{s1}}{V_{r1,s1}^{0}}d{\dot{z}}_{r1}+c\cdot \Delta d{\dot{t}}_{r1,r2}+\Delta V_{s1}^{0}+\Delta other \end{array}$$

where Δ indicates differencing between receivers.

Further, after differencing between a selected reference satellite and other satellites, we have the double-differenced (DD) equations:

(32) $$\left\{ \begin{array}{ll} \nabla \Delta \rho +\nabla \Delta v_{\rho }= & \left(\frac{x_{r1}^{0}-x_{s1}}{R_{r1,s1}^{0}}-\frac{x_{r1}^{0}-x_{s2}}{R_{r1,s2}^{0}}\right)\nabla \Delta dx+\left(\frac{y_{r1}^{0}-y_{s1}}{R_{r1,s1}^{0}}-\frac{y_{r1}^{0}-y_{s2}}{R_{r1,s2}^{0}}\right)\nabla \Delta dy\\ & +\left(\frac{z_{r1}^{0}-z_{s1}}{R_{r1,s1}^{0}}-\frac{z_{r1}^{0}-z_{s2}}{R_{r1,s2}^{0}}\right)\nabla \Delta dz+\nabla \Delta R^{0}+\Delta other\\ \nabla \Delta D+\nabla \Delta v_{D}= & \left(\frac{{\dot{x}}_{r1}^{0}-{\dot{x}}_{s1}}{V_{r1,s1}^{0}}-\frac{{\dot{x}}_{r1}^{0}-{\dot{x}}_{s2}}{V_{r1,s2}^{0}}\right)\nabla d{\dot{x}}_{r1}+\left(\frac{{\dot{y}}_{r1}^{0}-{\dot{y}}_{s1}}{V_{r1,s1}^{0}}-\frac{{\dot{y}}_{r1}^{0}-{\dot{y}}_{s2}}{V_{r1,s2}^{0}}\right)\nabla d{\dot{y}}_{r1}\\ & +\left(\frac{{\dot{z}}_{r1}^{0}-{\dot{z}}_{s1}}{V_{r1,s1}^{0}}-\frac{{\dot{z}}_{r1}^{0}-{\dot{z}}_{s2}}{V_{r1,s2}^{0}}\right)\nabla d{\dot{z}}_{r1}+\nabla \Delta V^{0}+\Delta other \end{array}\right.$$

where ∇ indicates differencing between satellites.

Generally, for one GNSS system, two receivers, and *s* satellites, using only pseudo-range and Doppler observations, the SD model has 2×s differenced observations and the DD model has 2×(s−1) differenced observations. The DD model seems to have a little advantage in computational cost. However, since such differencing will change matrix R, the computational cost difference between the two models using IF needs further study. Assuming s=3, we have matrix R of the undifferenced model as Equation (33). Then matrix R of the SD and DD models is as Equations (34) and (35), respectively. It can be seen that the DD model will transform R to nondiagonal.

(33) $$R=\left[\begin{array}{cccccc} R_{r1,s1} & & & & & \\ & R_{r1,s2} & & & & \\ & & R_{r1,s3} & & & \\ & & & R_{r2,s1} & & \\ & & & & R_{r2,s2} & \\ & & & & & R_{r2,s3} \end{array}\right]$$

(34) $$R=\left[\begin{array}{ccc} R_{r1,s1}+R_{r2,s1} & & \\ & R_{r1,s2}+R_{r2,s2} & \\ & & R_{r1,s3}+R_{r2,s3} \end{array}\right]$$

(35) $$R=\left[\begin{array}{cc} R_{r1,s2}+R_{r2,s2}+R_{r1,s1}+R_{r2,s1} & R_{r1,s1}+R_{r2,s1}\\ R_{r1,s1}+R_{r2,s1} & R_{r1,s3}+R_{r2,s3}+R_{r1,s1}+R_{r2,s1} \end{array}\right]$$

### 6.2. Test Settings

Our test used four GNSS datasets collected by two receivers. One receiver (named master) was fixed on the ground, and the other (named rover) was mounted on a van. The major information about the datasets is shown in Table 10.

The data-processing program was implemented in C++ language, with Eigen 3 as the linear algebra library [27]. The hardware platform was No. 11 in Table 7. The CV dynamic model in use was Equation (36), where $t_{0}$ is the epoch interval. The SD model corresponds to factor combination BAB, and the DD model corresponds to factor combination BAC. The filters contained six (three for position and three for velocity) or 10 (the six just mentioned, plus two for clock offsets and two for clock drifts) parameters when using the DD or SD model, respectively. Pseudo-range and Doppler observations were used. By using timing tools offered by the operating system, the running duration of each filter update cycle was collected.

(36) $$\left[\begin{array}{c} x_{k+1}\\ y_{k+1}\\ z_{k+1}\\ {\dot{x}}_{k+1}\\ {\dot{y}}_{k+1}\\ {\dot{z}}_{k+1} \end{array}\right]=\left[\begin{array}{cc} E_{3\times 3} & t_{o}\cdot E_{3\times 3}\\ 0 & E_{3\times 3} \end{array}\right]\left[\begin{array}{c} x_{k}\\ y_{k}\\ z_{k}\\ {\dot{x}}_{k}\\ {\dot{y}}_{k}\\ {\dot{z}}_{k} \end{array}\right]$$

### 6.3. Test Results and Analysis

**Figure 5:**
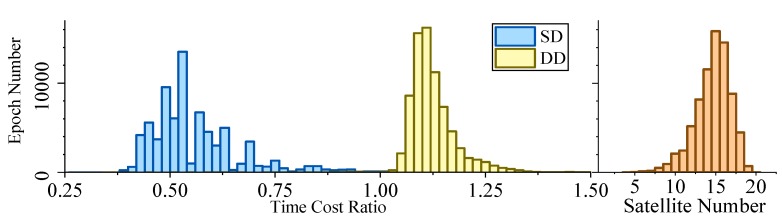
Time–cost ratio and satellite number histogram of dataset 1.

**Figure 6:**
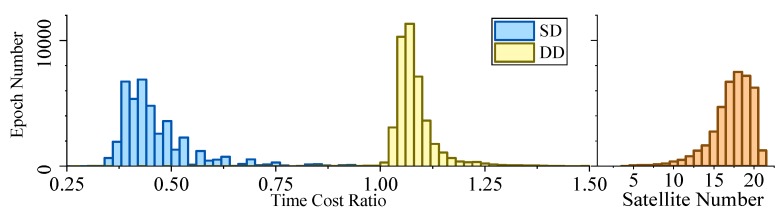
Time–cost ratio and satellite number histogram of dataset 2.

**Figure 7:**
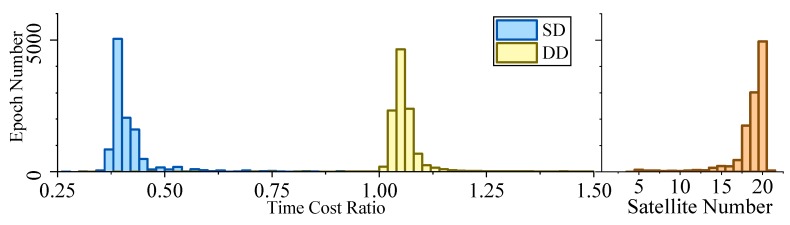
Time–cost ratio and satellite number histogram of dataset 3.

**Figure 8:**
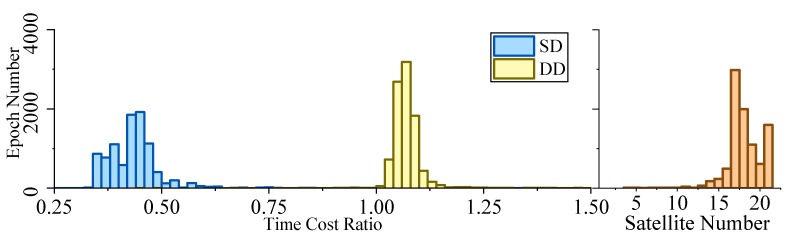
Time–cost ratio and satellite number histogram of dataset 4.

**Figure 9:**
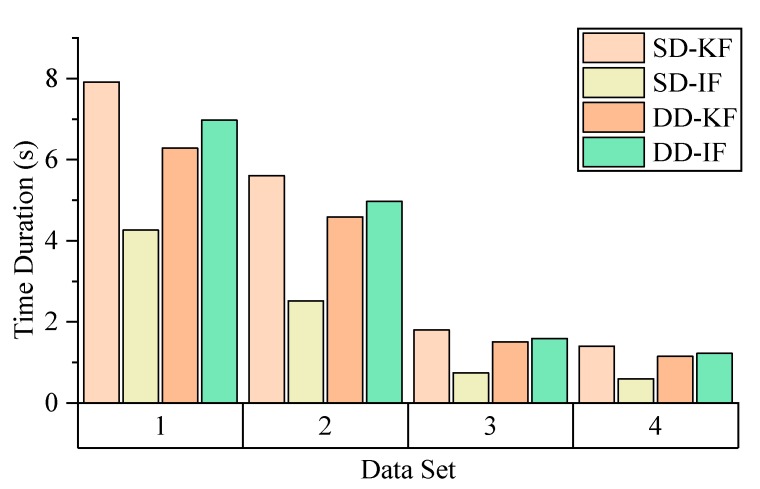
Overall time cost.

Figure 5, Figure 6, Figure 7 and Figure 8 demonstrate the distributions of the time duration ratio $t_{IF}/t_{KF}$ and the satellite number in the filter update step of each epoch, where $t_{IF}$ and $t_{KF}$ are the total durations of filter update procedures of the IF and KF, respectively. Figure 9 shows the total filter update time durations.

From the plots above, it can be seen that:

1. From Figure 5 to Figure 9, when the SD model is used, the IF takes about only 0.375–0.5 times the time duration of the KF, but when the DD model is used, this ratio is about 1.1. The average number of satellites in each dataset ranged from 15 to 20. Using that range and the number of parameters mentioned at the beginning of this section, we can read from Figure 4 that the predicted time–cost ratio ranges from 0.3 to 0.5 for the SD model, and from 1 to 1.05 for the DD model, which has good consistency with the test result.
2. From Figure 5 to Figure 8, the plots of time duration ratios and number of satellites have some dependency. This can be seen most easily from dataset 3, which has the most concentrated satellite number and time duration ratio distributions among all four.
3. From Figure 9, for the overall time duration, the KF runs obviously faster with the DD model than with the SD model, which can be attributed to fewer parameters and observations. The IF runs much faster with the SD model than with the DD model, which is consistent with our analysis that the computational advantage of IF over KF comes mostly from the assumption that matrix R is diagonal.

## 7. Conclusions

In this paper, we studied the computational differences between KF and IF, considering computational optimization specified by characteristics of different GNSS-related positioning and navigation algorithms, and the practicality of the results on various hardware platforms.

The major contributions and conclusions of this work are as follows:

1. Algorithm-specified optimizations of the IF were abstracted from GNSS-related positioning and navigation algorithms and tested theoretically. Among all three tested factors, the observation model was shown to impact the computational complexity of the IF the most. Also, the dynamic model and the solution requirement (whether epoch solutions are needed) were shown to influence the complexity.
2. Performance differences between the KF and IF in specific GNSS-related scenarios were studied. The IF did not perform better than the KF for algorithms with differencing between satellites or when using IMU data, while it seems to be a good alternative to the KF with better computational efficiency for GNSS-only algorithms with independent observations, especially those with fewer parameters, e.g., code-SDGNSS.
3. Differences between hardware platforms were studied to quantify the maximum error of applying our works on different platforms. Fifteen computers covering different CPU series from Intel and Advanced Micro Devices (AMD), operating systems of different versions of Windows and Linux, and desktop PCs, laptops, workstations, and servers were tested. The results show that the maximum error of using our calculated computational cost ratios to approximate those of other platforms is less than 10%, which guarantees the practicality of our conclusions on most X64 platforms. However, other platforms such as X86 and ARM still need further study.
4. SD and DD GNSS algorithms with the constant velocity dynamic model were chosen as case studies to study the performance differences between the KF and IF in real data-processing scenarios. The results show that the SD-KF model is, on average, 22% slower than the DD-KF model, which is consistent with it having slightly more parameters. However, the SD-IF model not only runs much faster than the DD-IF model (an average of 49% faster) but it also runs much faster than two KF models (average 55% and 45% faster than the SD-KF and DD-KF models, respectively).

## Figures and Tables

**Table 1:** Filter factors. DGNSS: GNSS difference positioning, DDGNSS: double-differenced DGNSS, GNSS: Global Navigation Satellite System, INS: inertial navigation system, PPP: precise point positioning.

Factors	Available Options	Examples
1. Solution requirements	A. Only the final solution based on all data is required.	PPP with static position parameters
	B. Epoch solutions are required.	Any dynamic positioning and navigation algorithm
2. Dynamic models	A. Dynamic model is fixed (F and W used in each epoch are the same).	PPP and DGNSS
	B. Dynamic model is not fixed, but matrix F is a diagonal matrix.	
	C. Dynamic model is not fixed, and matrix F is not a diagonal matrix.	GNSS/INS loosely or tightly coupled integrated navigation algorithms
3. Observation models	A. Observation model is fixed (B and R used in each epoch are the same).	
	B. Observation model is not fixed, but matrix R is a diagonal matrix.	Undifferenced and single-differenced GNSS algorithms
	C. Observation model is not fixed, and matrix R is not a diagonal matrix.	DDGNSS algorithms and GNSS/INS integrated navigation algorithms

**Table 2:** Optimization of the information filter (IF) using different factor options. Factors and options are from Table 1. Optimizations are summarized from previous discussion about each factor in Section 3.2.

Factors	Options	Optimizations
1	A	Equation (13) is omitted in the filter update procedure.
	B	None
2	A	$F^{-1}W$ in Equation (8) and $F^{-1}$ in Equation (9) are calculated beforehand and omitted in the filter update procedure.
	B	Calculation of $F^{-1}$ in Equations (8) and (9) gets easier and faster.
	C	None
3	A	$B^{T}R^{-1}B$ in Equation (11) and $B^{T}R^{-1}$ in Equation (12) are calculated beforehand and omitted in the filter update procedure.
	B	Calculation of $R^{-1}$ in Equations (11) and (12) gets easier and faster.
	C	None

**Table 3:** Scalar operation number polynomials of matrix operations.

Matrix Operations	Scalar Operations		
	Addition	Multiplication	Division
Addition ($A_{x\times y}+B_{x\times y}$)	xy	0	0
Multiplication ($A_{x\times y}\cdot B_{y\times z}$)	xyz	xyz	0
Inversion ($LU=A_{x\times x}$)	$\frac{5}{3}x^{3}+\frac{3}{2}x^{2}-\frac{1}{6}x$	$\frac{5}{3}x^{3}+\frac{1}{2}x^{2}-\frac{1}{6}x$	$x^{2}$
Inversion ($LDL^{T}=A_{x\times x}$)	$\frac{4}{3}x^{3}+\frac{1}{2}x^{2}+\frac{7}{6}x$	$\frac{4}{3}x^{3}+x^{2}+\frac{5}{3}x$	$\frac{1}{2}x^{2}+\frac{3}{2}x$

**Table 4:** Polynomial coefficients of scalar addition operation. IF: information filter, KF: Kalman filter.

Filter	Factors	Options	Polynomial Coefficients								
			$t^{3}$	$t^{2}n$	$tn^{2}$	$n^{3}$	$t^{2}$	tn	$n^{2}$	t	n
KF	AAA		3	3	2	4/3	3	2	3/2	1	13/6
IF	AAA		14/3				9/2	1		5/6	
	1	B-A	4/3				3/2			7/6	
	2	B-A	1								
		C-A	8/3				3/2			–1/6	
	3	B-A		1	2						
		C-A		1	2	4/3			1/2		7/6

**Table 5:** Polynomial coefficients of scalar multiplication operation.

Filter	Factors	Options	Polynomial Coefficients								
			$t^{3}$	$t^{2}n$	$tn^{2}$	$n^{3}$	$t^{2}$	tn	$n^{2}$	t	n
KF	AAA		3	3	2	4/3	1	2	1		5/3
IF	AAA		14/3				3/2	1		–1/6	
	1	B-A	4/3				2			5/3	
	2	B-A	1								
		C-A	8/3				1/2			–1/6	
	3	B-A		1	2						
		C-A		1	2	4/3			1		5/3

**Table 6:** Polynomial coefficients of scalar division operation.

Filter	Factors	Options	Polynomial Coefficients								
			$t^{3}$	$t^{2}n$	$tn^{2}$	$n^{3}$	$t^{2}$	tn	$n^{2}$	t	n
KF	AAA								1/2		3/2
IF	AAA						1				
	1	B-A					1/2			3/2	
	2	B-A								1	
		C-A					1				
	3	B-A									1
		C-A							1/2		3/2

**Table 7:** Tests of scalar operation time cost with different hardware platforms. CPU, central processing unit; OS, operating system.

No.	CPU	OS	Scalar Operation Time Cost (second)		
			dtA-dtEmpty	dtM-dtEmpty	dtD-dtEmpty
1	Intel I7-7700	Windows 10	2.043	2.042	85.225
2	Intel I7-7700	Windows 7	2.067	2.073	86.089
3	Intel I5-7300HQ	Windows 10	2.504	2.482	103.258
4	Intel I5-5350U	Ubuntu 16.04	1.265	1.265	107.148
5	Intel I5-4300U	Windows 7	2.363	4.895	220.207
6	Intel I5-4200H	Windows 8.1	1.183	2.384	106.244
7	Intel I3-3240	Windows 7	2.677	4.599	197.717
8	Intel I3-3110M	Ubuntu 16.04	1.658	3.328	153.334
9	Intel I3-2350M	Windows 7	1.784	3.461	169.764
10	Intel I3-2350M	Ubuntu 16.04	2.299	3.261	173.223
11	Xeon Silver 4114	Windows 10	3.133	3.147	132.182
12	Xeon E5-2609v3	Windows Storage Server 2012R2	2.366	5.169	243.789
13	Xeon E5-1620v4	Windows 7	1.026	1.025	89.846
14	AMD A10-5750M	Windows 10	4.143	4.126	402.132
15	AMD A6-5345M	Windows 7	5.066	5.100	492.969

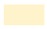
: Servers, workstations, and PC; 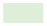: laptops; 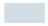: industrial tablet. dtA: time cost of addition; dtM: time cost of multiplication; dtD: time cost of division; dtEmpty: time cost of empty loop.

**Table 8:** Compositions and numbers of parameters and observations in GNSS algorithms. ZTD, zenith troposphere delay. IFPPP: ionosphere-free precise point positioning, SDGNSS: single-difference GNSS, RTK: real-time kinematic, UPPP uncombined precise point positioning.

Algorithm	Number of Parameters ^1^										Number of Observations			
	Position Corrections	Velocity Corrections	Attitude Corrections ^2^	Wet Component of ZTD	Receiver Clock Offset	Receiver Clock Drift	Ionospheric Delay ^3^	Ambiguity	Gyroscope Bias	Accelerometer Bias	Pseudo Range	Doppler	Carrier Phase	Other Observations
IFPPP with static position parameter	3	0	0	1	4	0	0	*s*	0	0	*s*	0	*s*	0
IFPPP with dynamic position parameter	3	0	0	1	4	0	0	*s*	0	0	*s*	0	*s*	0
UPPP with static position parameter	3	0	0	1	4	0	*s*	2 × *s*	0	0	2 × *s*	0	2 × *s*	0
UPPP with dynamic position parameter	3	0	0	1	4	0	*s*	2 × *s*	0	0	2 × *s*	0	2 × *s*	0
Code-SDGNSS with CV	3	3	0	0	4	4	0	0	0	0	2 × *s*	2 × *s*	0	0
Code-DDGNSS with CV	3	3	0	0	0	0	0	0	0	0	2 × *s* − 8	2 × *s* − 8	0	0
RTK/INS loosely coupled	3	3	3	0	0	0	0	0	3	3	0	0	0	6 ^4^
RTK/INS tightly coupled	3	3	3	0	0	0	0	2 × *s* − 8	3	3	2 × *s* − 8	2 × *s* − 8	2 × *s* − 8	0
RTK/INS tightly coupled with all ambiguities fixed	3	3	3	0	0	0	0	0	3	3	2 × *s* − 8	2 × *s* − 8	2 × *s* − 8	0
Code-DDGNSS/INS tightly coupled	3	3	3	0	0	0	0	0	3	3	2 × *s* − 8	2 × *s* − 8	0	0
IFPPP/INS tightly coupled	3	3	3	1	4	4	0	*s*	3	3	*s*	*s*	*s*	0

^1^ Tropospheric gradients are not included; ^2^ These parameters are elements of the misalignment angle rotation vector; ^3^ Pseudo-range and carrier-phase observations of two frequencies of the same satellite share the same ionospheric delay parameter; ^4^ These observations include position and velocity error observations.

**Table 9:** Number of parameters, number of observations, and factor combination of GNSS algorithms. CV: constant velocity.

Algorithms	Combinations	Number of Parameters	Number of Observations
IFPPP with static position parameter	AAB	s+8	2×s
IFPPP with dynamic position parameter	BAB	s+8	2×s
UPPP with static position parameter	AAB	3×s+8	4×s
UPPP with dynamic position parameter	BAB	3×s+8	4×s
Code-SDGNSS with CV	BAB	14	4×s
Code-DDGNSS with CV	BAC	6	4×s−16
RTK/INS loosely coupled	BCC	15	6
RTK/INS tightly coupled	BCC	2×s+9	6×s−24
RTK/INS tightly coupled with all ambiguities fixed	BCC	15	6×s−24
Code-DDGNSS/INS tightly coupled	BCC	15	4×s−16
IFPPP/INS tightly coupled	BCC	s+24	3×s

**Table 10:** Hardware information and test settings. IMU: inertial measurement unit.

**Master**	Antenna Type:	Novatel GPS-704-X	**Typical Test Environment**		Urban
	Receiver Type:	Novatel ProPak6	**City**	Dataset 1:	Huantai, Shandong
**Rover**	Antenna Type:	Novatel GPS-704-X		Dataset 2:	Huantai, Shandong
	Receiver Type:	Novatel ProPak6		Dataset 3:	Nanjing, Jiangsu
	IMU Type:	Novatel SPAN-LCI		Dataset 4:	Nanjing, Jiangsu
	Rover Type:	Van	**Time and Duration**	Dataset 1:	2016.11.13 08:37–12:50
**Typical Baseline Length**		4 km		Dataset 2:	2016.11.13 14:02–16:23
**GNSS Used**		GPS/BDS		Dataset 3:	2016.07.26 13:50–14:29
**Sampling Rate (second)**		0.2		Dataset 4:	2016.07.26 14:59–15:31
